# Effect of propolis on mood, quality of life, and metabolic profiles in subjects with metabolic syndrome: a randomized clinical trial

**DOI:** 10.1038/s41598-023-31254-y

**Published:** 2023-03-17

**Authors:** Sana Sadat Sajjadi, Mohammad Bagherniya, Davood Soleimani, Mansour Siavash, Gholamreza Askari

**Affiliations:** 1 Nutrition and Food Security Research Center and Department of Community Nutrition, School of Nutrition and Food Science, Isfahan University of Medical Sciences, Isfahan, Iran; 2 Anesthesia and Critical Care Research Center, Isfahan University of Medical Sciences, Isfahan, Iran; 3 Research Center of Oils and Fats, Kermanshah University of Medical Sciences, Kermanshah, Iran; 4 Nutritional Sciences Department, School of Nutrition Sciences and Food Technology, Kermanshah University of Medical Sciences, Kermanshah, Iran; 5 Isfahan Endocrine and Metabolism Research Center, Isfahan University of Medical Sciences, Isfahan, Iran

**Keywords:** Metabolic syndrome, Clinical trial design

## Abstract

Metabolic syndrome (MeS) is a common multifaceted disorder. Plants contain antioxidant bioactive compounds, which are beneficial to improve the health condition of patients with MeS. Propolis is a hive natural product that is composed of various constituent. We aimed to assess the effects of Iranian propolis as a natural and safe agent on indicators of MeS, quality of life and mood status in individuals with MeS. In total, 66 interested eligible patients recruited to the present study. Participants were randomly assigned to consume a tablet at dose of 250 mg of propolis extract, twice daily for 12 weeks or placebo. Propolis supplementation could lead to a significant reduction in waist circumference (WC), increase in physical functioning, general health and the overall score of SF-36 compared with placebo group (P-value < 0.05). However, no significant differences were observed regarding other anthropometric indices and biochemical parameters between two groups (P-value > 0.05). The current study indicated that propolis can be effective in decreasing WC and improving physical health and quality of life, while had no significant effects on other components of MeS among subjects with this syndrome.

* Clinical trials registration* Iran Registry of Clinical Trials.ir IRCT20121216011763N49, registration date 23/12/2020.

## Introduction

Metabolic syndrome (MeS) is a common multifaceted disorder. It is defined by pathological criteria including abdominal obesity, hypertension, impaired fasting blood glucose, and dyslipidemia^1,2^. People with MeS are at risk for various comorbidities such as cardiovascular diseases, cancers, polycystic ovary syndrome, and type 2 diabetes mellitus^3–5^. Also, there is a strong association between MeS and the development of oxidative stress and inflammatory condition^6^. MeS is regarded as one of the health concerns worldwide. Reports have shown that MeS imposes a great financial burden on health care system and its risk factors can lead to reduce mental and physical heaths and quality of life in individuals^7^. Data have indicated that number of people who suffer from this disorder. In a meta-analysis performed by Tabatabaei-Malazy et al., the prevalence of MeS in Iran based on the National Cholesterol Education Program Adult Treatment Panel III (NCEP ATP-III) criteria was reported 38.3%^8^. The etiology of MeS is not completely well understood yet. However, abdominal obesity and insulin resistance play a significant role on MeS pathogenesis. It has been reported that the main treatment approaches of MeS comprise lifestyle modification including dietary changes and increased physical activity^9,10^. However, it appears that these approaches may be unsuccessful due to poor compliance in a long term^11^. Recently, several clinical studies have shown potential effects of nutraceuticals and plant products in reduction of chronic disease complications and control MeS components. Plants contain antioxidant bioactive compounds, which are beneficial to improve the health condition of patients with MeS^12^.

Propolis is a hive natural product with various constituents. It has been recognized that different kinds of propolis contain active components including, phenolic acids, terpenes, amino acids, vitamins, numerous essential metals, and elements. Chemical composition of propolis is varied by bee species, plant origin, region of collection and, climate affect. Propolis is made by various bee species (Apis mellifera, stingless bees Meliponini and others) which play a major role on constituents of propolis^13–17^. In the past, propolis has been used as a folk medicine in treatment of infections and wounds^18^. Nowadays, propolis is prescribed as a popular dietary supplement to promote the body's health. Recently, propolis has been suggested to have various biological properties, including anti-tumor, antioxidant, antibacterial, anti-atherogenic, and anti-inflammatory activities^19–23^. Several animal and human studies have supported that propolis is effective in improving blood pressure, regulating glucose and lipid metabolisms, and enhancing the immune system function^24–30^. However, some investigations show inconsistent results. We observed that heterogeneity results for glycemic indices and lipid profiles are high. It might due to difference in form, dosage of used propolis, and duration of study. Instant to, result of a meta-analysis included six trials revealed that propolis significantly improved fasting plasma glucose, but did not effect on serum insulin and homeostasis model assessment-insulin resistance (HOMO-IR)^31^. A clinical study reported that Iranian propolis intake (1000 mg/d) in diabetic patients for 90 days significantly reduced HOMO-IR, inflammatory biomarkers and also increase level of high-density lipoprotein cholesterol (HDL-C) compared with controls^32^. However, a recent meta-analysis found that propolis supplementation alone or along with other components reduced serum fasting blood glucose, (FBG), hemoglobin A1c, and insulin, but had no effect on HOMO-IR and lipid profiles^33^. Also, a clinical study demonstrated that propolis intake with a dose of 500 mg/day for four months had no significant effect on lipid profiles and glycemic indices among patients with nonalcoholic fatty liver disease (NAFLD)^34^. Moreover, it is important to mention that most of these studies have evaluated the effect of propolis consumption on patients with type 2 diabetes.

To our knowledge, no randomized clinical trial has not been directly measured the effects of propolis on metabolic factors in patients with MeS. Therefore, we aimed to assess the effects of Iranian propolis as a natural and safe agent on indicators of MeS, quality of life and mood status in individuals with MeS.

## Materials AND methods

### Study design

The current study is a prospective, parallel, randomized, double-blind, placebo-controlled clinical trial. The study was conducted on subjects with MeS referred to an endocrine and metabolism research center and an outpatient clinic affiliated to Isfahan University of Medical Sciences, Isfahan, Iran. The ethics committee of Isfahan university of medical science reviewed and approved the study protocol (ID: IR.MUI.RESEARCH.REC1399.595). The clinical trial was registered at the Iranian Registry of Clinical Trial (Code: IRCT20121216011763N49). All methods were conducted based on the approved study plan, as well as with relevant guidelines and regulations.

### Participants

After evaluation the recorded information in the patient files based on inclusion criteria, in total, 66 interested eligible patients recruited to the present study through telephone calls and direct invitation from February 2021. We informed all individuals about study purpose and procedures. Written informed consent was signed by all participants before their participation in the study. The inclusion criteria consisted the following: Individual who had MeS when they met at least three or more of NCEP ATP-III criteria^35^: fasting plasma glucose ≥ 100 mg/dL, triglyceride (TG) ≥ 150 mg/dL HDL-C < 40 mg/dL in men or < 50 mg/dL in women, waist circumference (WC) ≥ 102 cm in men or ≥ 88 cm in women, blood pressure ≥ 130/85 mmHg, adults 20–60 years old, having the ability to read and write, a willingness to participate in the study and no change in type and dosage of lipid-lowering, hypotensive or hypoglycemic drugs over the past three months. The exclusion criteria consisted the following: pregnancy, breastfeeding, a sensitivity to bee products, use of smoking, alcohol and drugs, insulin therapy, patients who follow a weight loss diet or exercise program, a history of malignancy and cancer, type 1 diabetes, nephrotic syndrome, kidney and lung diseases, bile diseases and HIV. Patients who are unwilling to continue cooperating, become lactating and pregnant, have the sensitivity to propolis supplement, and suffer a specific disease during the study were withdrawn from follow-up.

### Randomization

All participants were randomly allocated to either propolis (n = 33) or placebo (n = 33) groups. Randomization was stratified according to sex (male vs. female), with the use of permuted block size of 4. The assignment sequences were provided by an independent statistician with the use of a random-number table and then were kept in opaque, sealed, numbered envelopes until the end of the eligibility criteria evaluation. Tablet containers were coded as A and B to order to allocation concealment. The study pharmacist coded tablet containers as A and B according to randomized list. Treatment assignments were concealed from researchers and all patients until the completion of data analyses, with the exception of the pharmacist.

### Interventions

Participants in intervention group were asked to consume a 350 mg propolis tablet (containing 250 mg of Iranian green propolis extract and 100 mg of safe and ineffective combination of microcrystalline cellulose) twice a day (a total of 500 mg Iranian green propolis extract/day) and the control group were asked to intake a same placebo tablet (containing 350 mg of microcrystalline cellulose) twice a day, one tablet before lunch and one tablet before dinner, for 12 weeks. Propolis sample was obtained from honey bee (Apis mellifera) colonies located in Rasht, a region in the north of Iran, in the summer season. First, propolis was ground and extracted with 70% ethanol at a ratio of 1:8. Then, the solution was sonicated by ultrasonic bath (Backer vCLEAN1-L20 Ultrasonic, Backer Co., Tehran, Iran) at 20 kHz and 35 degrees centigrade for 45 min. According to Bankova recommendation for chemical standardization of poplar propolis, total polyphenols content and total flavonoids content in poplar propolis tablets were measured using the spectrophotometric assay (JENWAY 7305, Bibby Scientific Ltd. Stone) based on the Folin–Ciocalteu reducing capacity and aluminum complex formation methods, respectively^36^. Each propolis tablet contains 90 mg gallic acid equivalent and 67 mg flavonoids. In this double-blind study, Pharmaceutical Company of Naghsh Jahan Ryhan (Isfahan, Iran), under the supervision of the study pharmacist produced placebo and propolis tablets with the same size, color, odor, form and packing. All the components of propolis and placebo tablets were manufactured totally the same except the bioactive component of propolis. The propolis and placebo groups conformed the same protocol (twice daily, before lunch and dinner). Investigators, participants, outcome assessors, researchers who measured anthropometric assessments, trained participant on how to fill out the questionnaires and laboratory staff and data analyzers were blinded to treatment assignment until the completion of data analyses.

At the beginning of the study, participants of both groups obtained healthy lifestyle recommendations. Regular use of supplements was reminded to patients through short message service and telephone call weekly and every two weeks, respectively. Also, their compliance to the study was evaluated by counting unused supplements at each visit. A 3-day food record (two weekdays and one weekend day) as the “gold standard” used for dietary intake assessment of each subject. The participants trained by a nutritionist who was unaware to the treatment allocation on how to complete 3-day food records at the beginning, the middle (weeks six), and the end of the intervention. Nutritionist IV software (First Databank Inc., Hearst Corp., San Bruno, CA, USA), which is adapted for Iranian foods was used to calculate the value of nutrition and calorie intake. A 3-day physical activity record (two weekdays and one weekend day) was used to assess physical activity of participants based on metabolic equivalent (MET)-h/day values.

### Measurements

Demographic characteristics, including age, sex, marital status, medical history, level of education, household status, and current drugs use were collected from each participant by completing a general questionnaire. Anthropometric variables of each patient including weight, height, body mass index (BMI), WC, and blood pressure were measured at the beginning and at the end of the study, after an overnight fast. Body weight was measured using a calibrated hand scale to the nearest 0.1 kg while the subjects wearing minimal clothing and no shoes (Seca, Germany). The scale was calibrated daily by a five-kilogram weight. Height without shoes in the standing position, while shoulders were in a normal position was measured using a stadiometer to the nearest 1 cm (Seca, Germany). Then, BMI was calculated as body weight in kilograms/body height in meters squared. WC was measured between the lower rib margin and the iliac crest at the end of normal exhalation, without any pressure to body surface using an inelastic tap, to the nearest 0.1 cm. an Experienced nutritionist measured blood pressure using a standard hand-held sphygmomanometer (ALPK2, Zhejiang, China; Datis Co, Tehran, Iran) over the right arm for each person, twice after the individuals had been sitting for 15 min. The average of two measurements was considered as the final blood pressure.

At the beginning and at the end of the intervention, a 10 cc venous blood sample was taken from participants after overnight 10–12 h fasting in the endocrine and metabolism research center laboratory (Esfahan, Iran). After separation of serum samples from whole blood, all aliquots were stored at −80 °C until biochemical analysis time. Serum insulin levels were measured using the (enzyme-linked immunosorbent assay) ELISA kit (Pars Azmoun kit, Tehran, Iran). FBG, concentration of serum cholesterol total (TC), TG, low-density lipoprotein cholesterol (LDL-C), and HDL-C were determined using the colorimetric technique by available standard kits (Pars Azmoun kit, Tehran, Iran). Also, LDL-C/HDL-C and cholesterol/HDL-C ratios were calculated. HOMO-IR was determined using the following formula: HOMA-IR = fasting glucose (mg/dl)*fasting insulin (μU/ml)/405^37^. Serum C-reactive protein (CRP) level was measured by the use of an immunoturbidimetric method (Bionik Diagnostic System, Tehran, Iran) and reagent kite (Pars Azmoun kit, Tehran, Iran).

### Quality of life

Quality of life was evaluated using 36-Item Short Form Health Survey (SF-36) by direct interview with participants at the beginning and the end of the intervention. SF-36 consisted of 36 questions that evaluate eight different domains of health. These eight domains can be summarized in to two components including physical component score and mental component score. Physical component score comprised domains of physical functioning, role limitations due to physical health, bodily pain and general health. Mental component score comprised domains of role limitations due to emotional problems, energy and fatigue, emotional well-being, and social functioning. The minimum and maximum scores in this questionnaire are zero and 100, respectively. Better quality of life is indicated by obtained higher scores^38^.

### Depression, anxiety and stress scale

The participants were asked to complete the Depression, Anxiety and Stress (DASS-21) questionnaire at the beginning and 12 weeks after the intervention. The DASS-21 as a tool to assess mental health, has 21 questions and three subscales (depression, stress, and anxiety). Each subscale of DASS-21 consist of seven questions^39,40^. Scores of the three subscales are calculated by summing the relevant responses and multiplying by two^41^. The minimum and maximum scores obtained in each subscale range between zero to 42, and lower score indicates a better situation of anxiety, depression and stress^42^.

### Dose of propolis

A study performed by Zakerkish et al. showed that the intake of 1000 mg/day of Iranian raw propolis supplement (the equivalent of 500 mg of propolis extract) for 3 months reduced HOMO-IR in patients with type 2 diabetes mellitus, without any side effects^43^. Also, Soleimani et al. revealed that the consumption of 500 mg/day of propolis extract improved hepatic steatosis and fibrosis among NAFLD patients^34^. Therefore, based on similar studies, propolis supplement was considered at a daily dose of 500 mg of propolis extract.

### Statistical analysis

All data were analyzed using SPSS 16 software (SPSS, Inc., Chicago, IL). The normal distribution of data was checked using Kolmogorov–Smirnov test and Q–Q plot. Data are presented as frequencies (percentage) for qualitative variables, mean (± SD) for normally distributed continuous data, or median (25th, 75th) for other variables. Within-group changes were assessed with the use of paired t test for normally distributed data, Chi-square test or Fisher's exact test for nominal variables, and Wilcoxon rank-sum test for other data. The between-group differences were assessed with the use of independent t test for normally distributed data, Chi-square test or Fisher's exact test for nominal variables, and Mann–Whitney U test for other data. Analysis of covariance (ACNOVA) (adjusted for baseline values) was used to detect any differences between the two groups at the end of the study. P-value < 0.05 was considered statistically significant. Sample size was calculated based alpha of 0.05, beta of 0.20 (power of 80%) and effect size of one unit. HOMA-IR was considered as a primary outcome variable. By considering 20% drop-out rate, in total, the sample size was estimated 30 subjects in each group^43^.

$$\mathrm{N}= \left(\frac{1+\varphi }{\varphi }\right)\left[\frac{{({Z}_{1-\frac{\alpha }{2}}+ {Z}_{1-\beta })}^{2}}{{\Delta }^{2}}+ \frac{{Z}_{1-\alpha /2}^{2}}{\varphi }\right]$$

### Ethical approval and consent to participate

The ethics committee of Isfahan university of medical science reviewed and approved the study protocol (ID: IR.MUI.RESEARCH.REC1399.595). The clinical trial was registered at the Iranian Registry of Clinical Trial (Code: IRCT20121216011763N49). Written informed consent was signed by all participants before their participation in the study.

## Results

**Figure 1:**
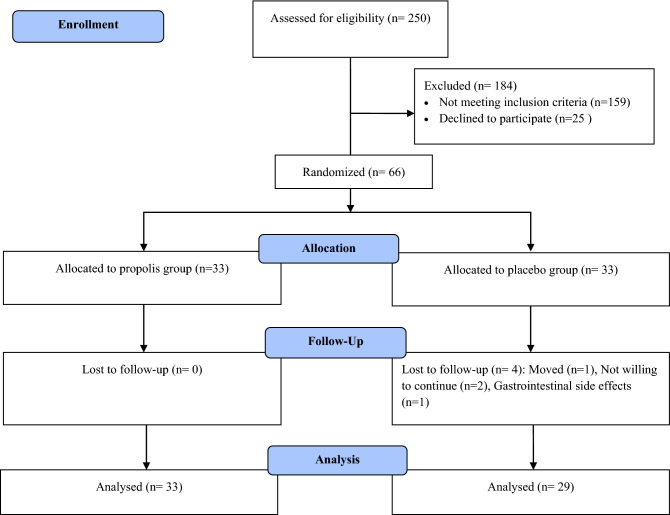
Flow chart of study participants.

Among the 66 participants with metabolic syndrome who enrolled in the clinical trial, 62 participants completed the trial (n = 33 in the propolis and n = 29 in the placebo, groups). During the intervention, four subjects from the placebo group were excluded due to move (n = 1), gastrointestinal side effects (n = 1), and not willing to continue (n = 2) (Fig. 1).

Table 1 shows baseline characteristics of participants who completed the study in both groups. The mean (± SD) of age in propolis group was 54.27 ± 6.58 years and in placebo group was 53.86 ± 5.60 years (P-value = 0.794). Fifty-seven women (30 persons in the intervention group) and five men (three persons in the intervention group) completed the trial study. At baseline, no statistically significant differences were found between groups in terms of demographic characteristics, body weight, BMI, WC, systolic blood pressure, diastolic blood pressure, consumption of vitamin supplements, current medications, and the number of subjects who had current diseases. Based on 3-days food records, there were no statistically significant differences in total energy and nutrients intakes within or between two groups. Moreover, the mean (± SD) of physical activity did not change between two groups (0.13 ± 2.67 MET-h/day in the propolis group vs. −0.27 ± 2.93 MET-h/day in the placebo group, P-value = 0.241) (Table 2).

**Table 1:** General characteristic of study participants in propolis and placebo groups at baseline.

Variables	Propolis group (N = 33)	Placebo group (N = 29)	P-value
Sex, n (%)			
Female	30 (90.9)	27 (93.1)	0.99^#^
Age, year	54.27 ± 6.58	53.86 ± 5.60	0.794
Weight, kg	79.04 ± 8.61	82.01 ± 13.41	0.298
BMI, kg/m2	32.56 ± 4.13	34.03 ± 4.78	0.201
WC, cm	107.16 ± 7.88	109.82 ± 9.99	0.246
Systolic blood pressure, mmHg	12.83 ± 1.67	13.01 ± 1.62	0.663
Diastolic blood pressure, mmHg	8.37 ± 1.06	8.55 ± 1.20	0.551
Vitamin supplement, n (%)	2(6.1)	0(0)	0.494
Current disease			
Thyroid disease, n (%)	5(15.2)	3(10.3)	0.713^#^
Gastrointestinal disease, n (%)	10(30.3)	12(41.4)	0.363
Liver disease, n (%)	6(18.2)	6 (20.7)	0.803
Cardiovascular diseases, n (%)	6(18.2)	7(24.1)	0.565
Current medication			
Lipid-lowering medication, n (%)	29(87.9)	26(89.7)	0.99^#^
Sugar-lowering medication, n (%)	29(87.9)	26(89.7)	0.99^#^
Pressure-lowering medication, n (%)	19(57.6)	18(62.1)	0.719
Levothyroxine medication, n (%)	5(15.2)	3(10.3)	0.713^#^
Change weight, n (%)	9(27.3)	6(20.7)	0.546
Marital status, n (%)			0.570^#^
Married	29 (87.9)	25 (86.2)	
Divorced/widowed	4(12.1)	4(61.38)	
Household status, n (%)			0.510^#^
Supervisor/Self- supervisor	7(21.2)	7(24.1)	
Under supervision	26(78.8)	22(75.9)	
Educational level, n (%)			0.357^#^
Under-diploma/Diploma	30(91)	28(96.6)	
University	3(9.1)	1(3.4)	
History of various diseases n (%)	33 (100)	28 (96.6)	0.468^#^

Values are presented as mean ± standard deviation and frequencies (percentages). Abbreviation: BMI, body max index; WC, waist circumference. P-values were derived from the independent-sample t test for quantitative variables and Chi-square test for qualitative variables between the two groups. ^#^Fisher's exact test.

**Table 2:** Dietary intake and physical activity of study participants before and after of the intervention.

Variables	Groups	Before	After	Changes	P-value ^b^	P-Value ^#^
Energy, kcal/day	Propolis	1538.67 [1340.16–2107.91]	1539.74 [1249.38–1857.92]	−39.45 ± 417.11	0.662	0.659
	Placebo	1413.04 [1190.56–2139.44]	1393.78 [1122.37–1812.83]	16.44 ± 571.09	0.940	
Protein, g/day	Propolis	67.62 [56.78–99.34]	67.04 [51.89–91.16]	−1.69 ± 26.81	0.809	0.953
	Placebo	67.32 [58.17–85.48]	64.31 [50.96–83.26]	−2.07 ± 23.58	0.738	
Carbohydrates, g/day	Propolis	255.25 [204.02–362.55]	237.94 [189.43–293.95]	−10.48 ± 64.33	0.561	0.396
	Placebo	199.85 [159.82–330.28]	214.82 [157.82–309.91]	7.61 ± 100.46	0.787	
Fat, g/day	Propolis	42.38 [34.47–57.03]	42.57 [35.15–49.31]	−0.08 ± 16.86	0.623	0.739
	Placebo	43.24 [32.92–58.08]	41.5 [28.58–55.64]	−1.58 ± 18.36	0.854	
SFA, g/day	Propolis	13.30 [11.22–16.86]	13.48 [11.21–16.87]	1.30 ± 9	0.964	0.949*
	Placebo	17.02 [11.22–19.87]	14.14 [10.58–20.66]	0.27 ± 8.17	0.787	
MUFA, g/day	propolis	14.15 [12.15–18.61]	14.20 [11.96–15.46]	0.14 ± 6.95	0.741	0.362
	Placebo	15.17 [11.66–20.82]	13.98 [9.98–19.52]	−1.51 ± 7.21	0.496	
PUFA, g/day	Propolis	8.18 [6.65–10.56]	7.65 [6.05–10.10]	−0.39 ± 3.83	0.598	0.952
	Placebo	8.12 [4.99–11.01]	7.35 [5.64–9.76]	−0.45 ± 3.84	0.922	
Cholesterol, mg/day	Propolis	200.19 [115.40–284.92]	198.35 [131.17–264.21]	14.77 ± 135.80	0.688	0.530*
	Placebo	187 [126.11–308.18]	148.85 [119.73–285.53]	38.59 ± 345.68	0.642	
Fructose, g/day	Propolis	19.12 [13.6–24.62]	16.58 [8.53–23.35]	−2.07 ± 9.11	0.437	0.334*
	Placebo	12.09 [7.05–17.68]	13.81 [7.17–19.80]	2.01 ± 13.27	0.596	
Magnesium, mg/day	Propolis	411.42 [270.56–566.36]	360.03 [285.64–468.90]	−23.21 ± 216.32	0.437	0.857
	Placebo	345.61 [248.72–442.09]	295 [219.87–394.40]	−32.34 ± 174.76	0.469	
Zinc, mg/day	Propolis	11.92 [7.63–14.34]	10.39 [8.39–13.35]	−0.26 ± 5.21	0.675	0.652
	Placebo	10.61 [8.47–12.91]	9.65 [6.65–12.87]	−0.84 ± 4.99	0.596	
Selenium, ug/day	Propolis	102.29 [84.51–128.74]	95.50 [77.82–122.70]	2.92 ± 48.13	0.662	0.334*
	Placebo	84.32 [71.35–128.31]	80.91 [65.29–103.38]	−12.63 ± 50.22	0.198	
Calcium.mg/day	Propolis	1164.55 [578.14–1998.19]	1062.07 [550.04–1434.69]	−221.18 ± 1082.62	0.272	0.668
	Placebo	1064.78 [562.33–1364.04]	815.97 [439.13–1284.16]	−113.59 ± 846.16	0.496	
Vitamin C, mg/day	Propolis	117.82 [53.85–228.71]	84.45 [66.33–157.08]	−24.89 ± 106.55	0.313	0.822
	Placebo	95.69 [67.65–187.27]	80.79 [42.93–131.97]	−30.50 ± 86.27	0.122	
Fiber, g/day	Propolis	36.38 [20.90–55.55]	31.40 [21.80–45.30]	−2.93 ± 22.69	0.448	0.913
	Placebo	29.88 [19.24–37.12]	21.34 [16.28–37.65]	−3.53 ± 19.42	0.496	
Physical activity (MET-h/week)	Propolis	32.56 ± 2.58	32.69 ± 3.07	0.13 ± 2.67	0.781^a^	0.241*
	Placebo	34.62 ± 3.79	34.35 ± 2.46	−0.27 ± 2.93	0.624^a^	

Values are presented as mean ± standard deviation or median [interquartile range]. Abbreviation: SFA, saturated fatty acids; MUFA, monounsaturated fatty acids; PUFA, polyunsaturated fatty acids. *P-value were derived from Mann–Whitney U test. ^#^P-value were derived from Independent Sample t test. ^b^P-value were derived from Wilcoxon rank-sum test .^a^P-value were derived from the Paired- sample t-test.

As reported in Table 3, propolis supplementation significantly reduced the mean weight, BMI, and systolic blood pressure in the propolis group (P-value = 0.016, P-value = 0.016, and P-value = 0.020, respectively). However, the differences were not significant between two groups (P-value = 0.550, P-value = 0.217, and P-value = 0.366, respectively). Also, there was a significant reduction in the mean WC compared with baseline value in the propolis group (P-value < 0.001) and this difference was statistically significant compared with the placebo group (P-value = 0.008). A significant reduction in serum concentrations of TC compared with baseline value was observed in the propolis group (P-value = 0.039). In addition, HDL-C level significantly reduced in both groups. However, we did not find any significant differences between two groups for serum levels of TC, HDL-C, LDL-C, CRP, insulin, FBG, TG, LDL/HDL ratio, cholesterol/HDL ratio, and HOMO-IR.

**Table 3:** The effects of propolis supplementation on anthropometric indices, blood pressure, inflammation status, glycemic parameters and lipid profiles in two groups.

Variables	Groups	Before	After	Changes	P-Value ^b^	P- value^#^
Weight, kg	Propolis	79.05 ± 8.61	77.97 ± 8.52	−1.08 ± 2.43	0.016	
	Placebo	82.02 ± 13.42	81.64 ± 13.08	−0.38 ± 1.98	0.310	0.550*
	P-Value	0.298				
BMI, kg/m2	Propolis	32.57 ± 4.13	32.12 ± 4.06	−0.45 ± 1	0.016	
	Placebo	34.03 ± 4.79	33.88 ± 4.73	−0.15 ± 0.84	0.348	0.217
	P-Value	0.201				
Waist circumference, cm	Propolis	107.17 ± 7.89	104.08 ± 6.96	−3.09 ± 4	< 0.001	
	Placebo	109.83 ± 9.99	109.66 ± 10.26	−0.17 ± 4.36	0.833	0.008
	P-Value	0.246				
SBP, mmHg	Propolis	12.83 ± 1.67	12.16 ± 1.23	−0.67 ± 1.58	0.020	
	Placebo	13.02 ± 1.63	12.67 ± 1.37	−0.34 ± 1.21	0.137	0.366
	P-Value	0.663				
DBP, mmHg	Propolis	8.38 ± 1.07	8.08 ± 0.91	−0.30 ± 1.41	0.237	
	Placebo	8.55 ± 1.21	8.26 ± 0.85	−0.29 ± 1.31	0.237	0.995
	P-Value	0.551	0.438			
LDL/HDL ratio	Propolis	2.06 ± 0.47	2.06 ± 0.32	−0.01 ± 0.42	0.934	
	Placebo	2 ± 0.57	2.11 ± 0.39	0.11 ± 0.48	0.211	0.296
	P-Value	0.631				
Cholesterol/HDL ratio	Propolis	3.66 ± 0.45	3.70 ± 0.44	0.05 ± 0.41	0.525	
	Placebo	3.60 ± 0.55	3.72 ± 0.44	0.12 ± 0.55	0.257	0.760
	P-Value	0.673				
TG, mg/dl	Propolis	239.97 ± 169.76	234.33 ± 144.94	−5.64 ± 212.18	0.880	
	Placebo	224.34 ± 129.58	288.34 ± 192	64 ± 225.16	0.137	0.215
	P-Value	0.688				
Insulin, μU/ml	Propolis	4.87 [3.73–8.81]	7.34 [3.88–8.88]	0.75 ± 3.69	0.357^a^	
	Placebo	5.38 [3.2–8.89]	6.37 [4.12–9.47]	0.64 ± 2.51	0.163^a^	0.890
	P-Value	0.849				
FBG, mg/dl	Propolis	126 [112.5–168.5]	123 [104–172]	−5.06 ± 57.11	0.381^a^	
	Placebo	123 [108–174.5]	133 [114–160]	0.48 ± 53.53	0.387^a^	0.197*
	P-Value	0.827				
HOMO-IR	Propolis	1.93 [1.22–2.88]	1.90 [1.29–3.05]	0.42 ± 2.33	0.662^a^	
	Placebo	1.57 [1.04–2.96]	1.84 [1.26–3.43]	0.33 ± 1.28	0.107^a^	0.805*
	P-Value	0.530				
TC, mg/dl	Propolis	168 [143.5–198]	141 [129–187]	−9.42 ± 34.98	0.039^a^	
	Placebo	180 [150.5–199.5]	162 [140–201.5]	−10.55 ± 31.41	0.106^a^	0.895
	P-Value	0.489				
HDL-C, mg/dl	Propolis	45 [39.5–57]	41 ^32,36–50^	−2.52 ± 14.42	0.022^a^	
	Placebo	50 [42.5–54.5]	44 [37.5–51.5]	−4.14 ± 8.65	0.003^a^	0.865*
	P-Value	0.259				
LDL-C, mg/dl	Propolis	93 [77.5–109.5]	79 [69.5–109]	−3.70 ± 37.73	0.114^a^	0.682*
	Placeo	94 [76–124.5]	90 [78–123]	−2.52 ± 29.03	0.406^a^	
	P-Value	0.899				
CRP, mg/l	Propolis	1.4 [0.2–2.65]	1.8 [0.25–4.3]	0.56 ± 3.59	0.084^a^	
	Placebo	1.8 [0.2–5.15]	2.8 [1–5.1]	−0.48 ± 7.23	0.838^a^	0.341*
	P-Value	0.329				

Values are presented as mean ± standard deviation and median [interquartile range]. Abbreviation: FBS, fasting blood sugar; HDL-C, high-density lipoprotein cholesterol; LDL-C, low-density lipoprotein cholesterol; HOMA-IR, homeostasis model of assessment-insulin resistance; CRP, C- reactive protein; TC, total cholesterol; SBD, systolic blood pressure; DBP, diastolic blood pressure; BMI, body mass index. * P-value were derived from Mann-Whiney U test. ^#^ P-value were derived from Independent t test. ^b^ P-value were derived from paired-sample t test. ^a^ P-value were obtained from Wilcoxon rank-sum test.

The effects of propolis supplementation on mood status and quality of life before and after of intervention are reported in Table 4. The results showed that the mean score of anxiety was significantly reduced in the propolis group (P-value = 0.023), however in compression with the placebo group the mean scores of anxiety, stress, and depression were not significant (P-value = 0.921, P-value = 0.071, and P-value = 0.250, respectively). After the intervention, the mean physical functioning (P-value < 0.001), general health (P-value < 0.001), and the total score of SF-36 (P-value < 0.001) was significantly increased in the propolis group compared with the placebo group. Moreover, energy fatigue domain was increased in the propolis group (P-value = 0.005), however this difference was not significant between two groups. There was a significant different between groups regarding bodily pain (P-value = 0.015) (Supplementary file 1).

**Table 4:** The effects of propolis supplementation on mood status and quality of life before and after of intervention.

Variables	Groups	Before	After	Changes	P-value ^a^	P-value ^#^
DASS-21						
Depression	Propolis	10.5 ± 8.94	9.39 ± 9.09	−1.94 ± 7.70	0.164	
	Placebo	11.93 ± 6.79	12.43 ± 10.57	0.44 ± 8.01	0.775	0.250
	P-value	0.494				
Anxiety	Propolis	13.13 ± 9.38	11.27 ± 7.78	−2.69 ± 6.36	0.023	
	Placebo	15.07 ± 9.16	12.71 ± 9.68	−2.52 ± 6.62	0.059	0.921
	P-value	0.421				
Stress	Propolis	15.63 ± 10.24	14.60 ± 9.49	−1.75 ± 9.04	0.282	
	Placebo	19.36 ± 10.24	21.21 ± 9.88	1.85 ± 5.87	0.113	0.071
	p-value	0.169				
SF-36						
physical functioning	Propolis	82.58 ± 15.82	91.36 ± 9.94	8.79 ± 12.75	< 0.001	
	Placebo	66.92 ± 18.85	70.17 ± 19.34	3.25 ± 12.97	0.188	< 0.001*
	P-value	< 0.001				
Role limitation due to emotion problem	Propolis	44.44 ± 43.83	57.58 ± 48.79	13.13 ± 55.86	0.165	
	Placebo	40.23 ± 45.76	42.53 ± 47.89	2.30 ± 53.4	0.056	0.440
	P-value	0.713				
Limit to physical	propolis	42.42 ± 46.56	53.03 ± 46.25	10.61 ± 51.93	0.352	0.401*
	Placebo	22.41 ± 39.16	27.59 ± 43.99	5.17 ± 51.05	0.186	
	P-value	0.071				
Energy fatigue	Propolis	59.70 ± 22.81	67.73 ± 17.05	8.03 ± 15.15	0.005	
	Placebo	50.69 ± 19.94	52.07 ± 19.62	1.38 ± 17.06	0.667	0.109
	P-value	0.105				
Emotional well being	Propolis	66 ± 19.49	68.61 ± 18.79	2.61 ± 12.93	0.255	
	Placebo	53.14 ± 20.20	54.38 ± 22.09	1.24 ± 17.31	0.702	0.222*
	P-value	0.013				
Social functioning	Propolis	72.35 ± 28.94	74.95 ± 27.24	2.61 ± 29.90	0.620	0.732
	Placebo	71.12 ± 29.71	76.72 ± 32.69	5.60 ± 38.61	0.441	
	P-value	0.870				
Bodily pain	Propolis	69.39 ± 33.69	76.74 ± 28.02	7.35 ± 38.56	0.282	
	Placebo	46.98 ± 33.68	50 ± 332.40	3.02 ± 30.60	0.6	0.015*
	P-value	0.011				
General health	Propolis	53.62 ± 19.06	68.94 ± 19.48	15.32 ± 19.83	< 0.001	
	Placebo	49.61 ± 15.17	50.13 ± 16.34	0.52 ± 15.46	0.858	< 0.001
	p-value	0.367				
Total	propolis	62.87 ± 15.36	73.02 ± 13.41	10.15 ± 11.33	< 0.001	
	Placebo	51.53 ± 15.93	54.16 ± 16.65	2.64 ± 11.37	0.222	< 0.001*
	p-value	0.006				

Values are presented as mean ± standard deviation. Abbreviations: Dass-21, the 21-Item Depression Anxiety and Stress Scale; SF-36; the 36-Item Short Form Health Survey. * P-value were derived from ANCOVA test with baseline values as the covariate. ^#^ P-value were obtained from Independent Sample t test. ^a^ P-value were derived from paired-sample t test.

## Discussion

The findings of this double-blind, randomized and placebo-controlled study demonstrated that daily intake of 500 mg Iranian propolis extract in subjects with MeS for 12 weeks could lead to a significant reduction in WC, increase in physical functioning, general health, and the overall score of SF-36. While, propolis supplementation had no effects on other anthropometric indices and biochemical parameters compared to the placebo group.

In the propolis group, compared with baseline, at the end of the study, a significant reduction was observed in anthropometric indices including WC, BMI, and body weight. Nonetheless, significant differences were observed between two groups for WC, but not regarding BMI and body weight. In three previous interventional studies that assessed the effects of propolis on WC measurement^44–46^, propolis had no considerable effects on WC. In addition, some clinical trials^23,27,34,43–46^ indicated that propolis consumption had no favorable effects on weight and BMI. However, a clinical trial showed that daily intake of 900 mg of raw propolis supplementation for 12 weeks in diabetic subjects could reduce weight and BMI^24^. Nevertheless, a previous study among healthy subjects reported that 1000 mg daily raw propolis consumption for 60 days significantly increased BMI and weight^47^. It seems that the controversies in outcomes of researches might be related to differences in form and amount of used propolis, the study population, duration of intervention or the effects of confounders such as change in dietary intakes. Although, finding of current meta-analysis showed that propolis had no significant effects on anthropometric indices^33^. In preclinical studies, several anti-obesity mechanisms for propolis are considered. For example, it has been shown that propolis had a role in expression of factors that involved in lipid metabolism. Propolis inhibits accumulation of visceral adipose tissues and weight gain through controlling factors such as, SREBP- 1, SREBP-2, and down-regulation of PPARγ protein in the adipocytes^48,49^. PPARα protein regulates genes associated with fatty acid degradation. The increase of PPARα protein in liver by propolis can lead to increase β-oxidation of fatty acids^50^. Furthermore, it has been suggested that propolis can prevent the absorption of fat from the intestines in animal models^29^.

We found that propolis supplementation for 12 weeks had no effects on FBG, serum insulin, and HOMO-IR among subjects with MeS. Consist with our study, the result of a trial indicated that intake 500 mg/day of propolis extract for four months did not improve FBG, serum insulin, and HOMO-IR in patients with NAFLD^34^. Data from two interventional studies reported that daily supplementation of 900 mg raw propolis during 18 weeks had no beneficial effects on FBG, serum insulin and Hemoglobin A1c among patients with type 2 diabetes mellitus^28,51^. Also, some interventional studies found no significant association between propolis and HOMO-IR and serum insulin^24,34,45,47,52^. Conversely, a clinical study indicated that propolis extract supplement at a daily dosage 1000 mg for 90 days improved glycemic parameters in diabetic subjects^43^. Also, other report showed that daily supplementation of 1500 mg raw propolis for eight weeks significantly reduced FBG, serum insulin, and HOMO-IR in diabetic patients^25^. A currently published meta-analysis conducted by Hallajzadeh et al. showed that propolis supplements lead to reduce FBG, Hemoglobin A1c, and serum insulin, but did not effect on HOMO-IR^33^. It has been proposed that bioactive components of propolis such as flavonoids can stimulate glucose uptake and up-regulate the expression of insulin‐sensitive glucose transporter (GLUT) 4 in skeletal muscle. Propolis and its derivatives also can down-regulate the expression of genes involved in gluconeogenesis such as, glucose‐6‐phosphatase enzyme, reduce gut glucose absorption, elevate glucose utilization by liver's cells and increase cellular insulin sensitivity^45,53,54^. It seems that in our study using low dose of propolis resulted in non-significant changes on glycemic indices. The biological activities of propolis are related to its chemical component. Reports have shown that agents such as, bee species, plant origin, region of collection and climate affect the chemical composition of propolis. Generally, the amounts of chemical composition of collected propolis from different parts of the world are various^55^. According to the Bankova classification, propolis is divided into Brazilian, Canarian, Chinese, poplar Egyptian and pacific types. Studies have shown that identified propolis in the temperate region has more caffeic acid phenethyl ester (CAPE)^56^. However, the major bioactive component of propolis collected in the tropical region is prenylated phenylpropanoids and diterpenes such as, Brazilian green propolis^57^. European propolis has more mount of polyphenolic component than Brazilian propolis. Furthermore, it has been proposed that different species of bees can influence on bioactive components of propolis. Several reports have indicated that propolis is produced by stingless bees and Apis mellifera from tropical countries has similar composition^58^. Different species of Apis mellifera impact on chemical component of propolis^59^. Studies have reported that different components of propolis lead to cause various pharmacology activities^60^. In our study, propolis sample were collected from honey bee (Apis mellifera) colonies located in Rasht, a region in the north of Iran, in the summer season. Each propolis tablet contains 90 mg gallic acid equivalent and 67 mg flavonoids. While, other studies included in the discussion section investigated effect of different kinds of propolis collected from different regions on fasting blood glucose (FBG), insulin levels. Thus, it seems that in addition to difference in doses, difference in bioactive component of propolis may play a role.

In our study, no significant effect was found on lipid profile after taking propolis in subjects with MeS. Our finding is in agreement with the results of two meta-analyses that indicated propolis supplementation had no effects on lipid indices^33,61^. Also, another study reported that propolis supplementation for four months did not improve components of lipid profile among patients with NAFLD^34^. In contrast, a previous study observed a significant increase in HDL-C after 1000 mg/day of raw propolis supplementation for 90 days^43^. Another study, conducted by Samadi et al. found that propolis supplementation could reduce serum levels of LDL-C and TC among diabetic patients^24^. A meta-analyses with five studies showed that propolis could reduce TG level and increase HDL-C level^62^. In this regard, some potential pathways have been suggested for the effects of propolis in modulating blood lipid. Propolis can reduce cholesterol accumulation in the macrophage through up-regulation of PPAR gamma, and liver X receptor. Also, propolis can cause reducing in the activity of HMG-COA reductase protein and increase the expression of ATP-binding cassette transporters (ABC) A1 and G1 genes in hepatocytes which related to cholesterol metabolism^63,64^. In addition, the blood TG lowering effect of propolis can be attributed to insulin-mediated lipoprotein lipase activity^65^.

In this study, we observed no difference in the concentration of serum CRP between two groups after propolis intake, which is accordance with the data from studies performed by Fukuda et al.^52^ and Mujica et al.^45^. However, two meta-analyses^33,66^ and one systematic review^67^ indicated that propolis supplementation improves inflammation status. The possible reasons for the effect of propolis on inflammation status might due to the difference in amount of used propolis and duration of intervention. It has been suggested that propolis can reduce the production of pro-inflammatory cytokines by inhibiting the expression of nuclear factor-kappa (NF-κB), Jun N-terminal Kinase (JNK), and cyclooxygenase 2. NF-κB is an essential transcription factor that involved in the expression of inflammatory gene. It has been shown that caffeic acid phenethyl ester (CAPE) derived from propolis reduces the inflammation process through the inhabitation of NF-κB activation and the degradation of NF-κB inhibitor^68,69^.

Some animal studies have shown a significant reduction in blood pressure following consumption of propolis through decreasing the tyrosine hydroxylase activity which involved in biosynthesis of catecholamine. The presence of antioxidant components in propolis might play a role in vasorelaxation through down-regulation of nicotinamide adenine dinucleotide phosphate oxidase (NOX) and increase nitric oxide synthase (NOS) activity^70^. Another mechanism has been shown that propolis reduces blood pressure through suppress Na + reabsorption in renal tubules by the reduction of insulin level^71^. The result of a study indicated that intake 1000 mg/d of propolis for two months could be beneficial on blood pressure among healthy volunteers with normotensive^72^. Nevertheless, our study reported that there was a significant improvement in systolic blood pressure in the propolis group, but no significant difference was shown in systolic blood pressure and diastolic blood pressure between two groups. In similar to, the data from a study conducted by Silveira et al. reported that propolis extract supplement at a dose of 500 mg/day with antihypertensive drugs for 12 months did not effect on blood pressure among patients with chronic kidney disease^73^. It seems that difference in dosage of used propolis and the study population may be reasons of our finding. In the study conducted by Silveira et al., participants were with chronic kidney disease and under treatment with antihypertensive drugs.

We observed that the mean score of anxiety reduced significantly in the intervention group. However, there was no significant difference in three subscales of DASS-21 including, stress, anxiety and depression between the two groups. In six weeks randomized trial, Miryan et al. found that daily supplementation of 900 mg propolis improved anxiety and quality of life among irritable bowel syndrome patients^44^. Some in vivo and in vitro studies have reported that antioxidant agents such as, terpenoids, aromatic and aliphatic components identified in essential oil of propolis have neuroprotective effects and play an important role in the improvement of cognitive functions^74^. It has been shown that hyperactivity of hypothalamic–pituitary–adrenal (HPA) in brain can lead to increase production of plasma cortisol and adrenocorticotropic hormone concentrations that influence on body physiological processes, as well as depression, stress and anxiety^75^. Aromatic carboxylic acids and terpenoids contain in propolis essential oil can lead to an improvement in anxiety behavior through increasing the activity of superoxide dismutase (SOD) enzyme, inhibiting the activity of lipid HPA axis in brain tissue^76^. Also, it has been suggested that propolis has antidepressant activity, which might be done through the modulation of HPA axis and increasing the expression of hippocampal glucocorticoid receptor (GR) in animal models^77^. According to the above statements, we expected that propolis supplement improve anxiety, stress and depression in the subjects with MeS. However, no significant association between propolis and mood was observed. So, it seems that higher dosages of propolis and longer durations of study may be needed to observe the significant association. It is important to note that our study was conducted during the coronavirus disease (COVID-19) pandemic that lead to cause a stressful condition in the general population. It has been reported that the prevalence of COVID-19 has adverse effects on mental health due to change in life style of most individuals. Most populations in the world experienced psychological problems including, stress, depression and anxiety during COVID-19 pandemic. In the present study, quality of life was evaluated in components of mental and physical health. We observed that propolis could improve physical health and as well as, the overall score of quality of life. This is consistent with the data from studies performed by Miryan et al. and Presicce et al. In the study of Presicce et al., Boswellia resin extract and propolis derived polyphenols consumption ameliorate quality of life in patients with diabetic mellitus after 90 days^78^. Evidence has been indicated that high reactive oxygen species levels in skeletal muscle reduce muscles force, elevate fatigue and disrupt the cellular functions. This condition can cause reduce the ability of body and eventually has a negative effect on quality of life. It has been shown that polyphenols components and CAPE identified in propolis reduce damage to muscle and improve physical performance through, inhibiting NF-κB signaling pathway and increasing the activity of antioxidant enzymes^79^.

Some limitations should be noted in our study. The effect of propolis alone could not be investigated, due to ethical issues. The main limitation of the current study is that it was conducted during the Covid-19 pandemic. Covid-19 has caused a stressful condition upon the major of individuals which has negative effects on different aspects of life. Probably, this tissue can distort our results. The strength points of our study included the following: firstly, the present study is a parallel randomized double-blind clinical trial that has been performed for the first time among subjects with MeS. Third, the control and intervention groups were matched in terms of baseline values.

## Conclusion

In total, the current study indicated that propolis as a natural, safe and available supplement can be effective in decreasing WC and improving physical health and quality of life, while had no significant effects on other components of MeS among subjects with this syndrome. So, further studies with higher dosages of propolis will be needed to explain the exact mechanisms theses favorable effects and draw a clear conclusion the association between propolis with components of MeS in subjects with MeS.

## Supplementary Information

Supplementary Information.

## Data Availability

The data analyzed during the current study are available from the corresponding author on a reasonable request.
