# Supplementary material for: Effect of propolis on mood, quality of life, and metabolic profiles in subjects with metabolic syndrome: a randomized clinical trial
Source: Sci Rep. 2023 Mar 17;13:4452. doi: 10.1038/s41598-023-31254-y (PMC10022550; doi:10.1038/s41598-023-31254-y)
Supplement: Supplementary file 1 — Supplementary Information. [file 41598_2023_31254_MOESM1_ESM.docx]

**Supplementary figure 1. Histogram plots of the quality of life in two groups, before and after intervention**

**
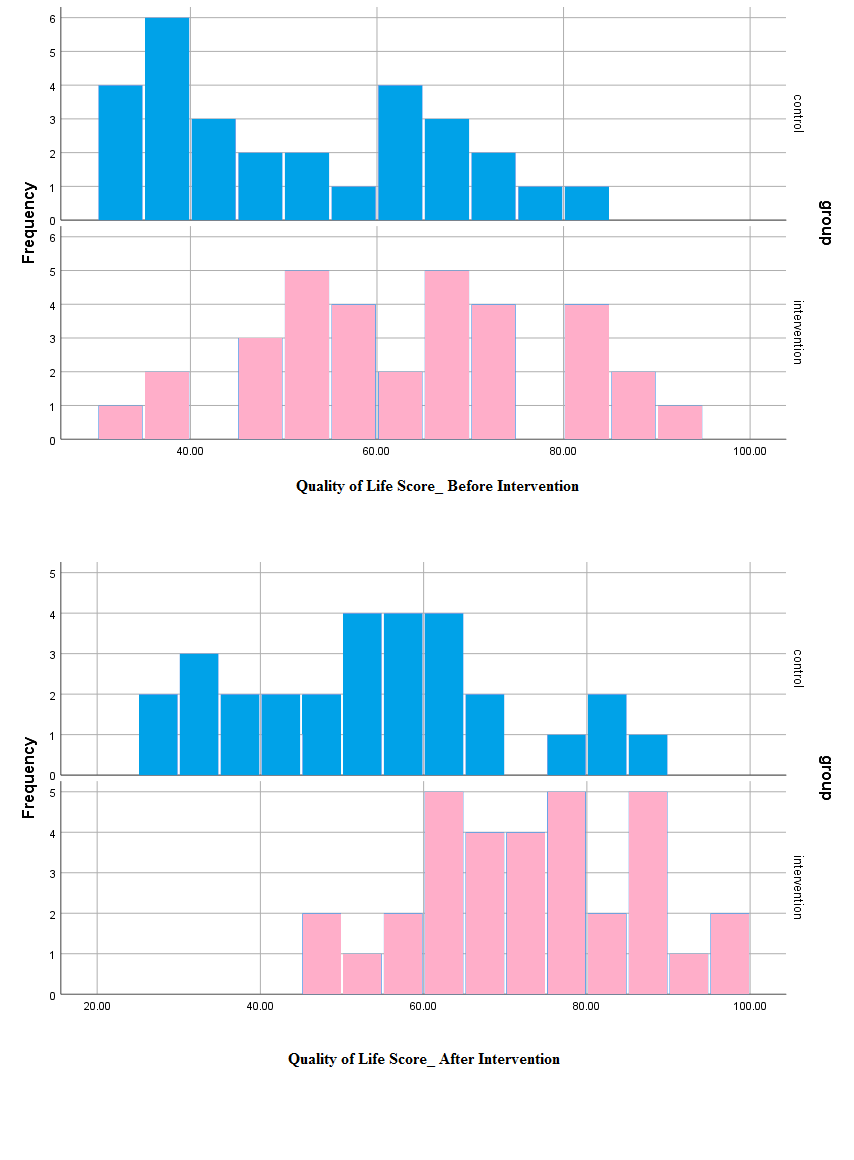
**

**Supplementary figure 2. Histogram plots of the DASS 21 score in two groups, before and after intervention**

**
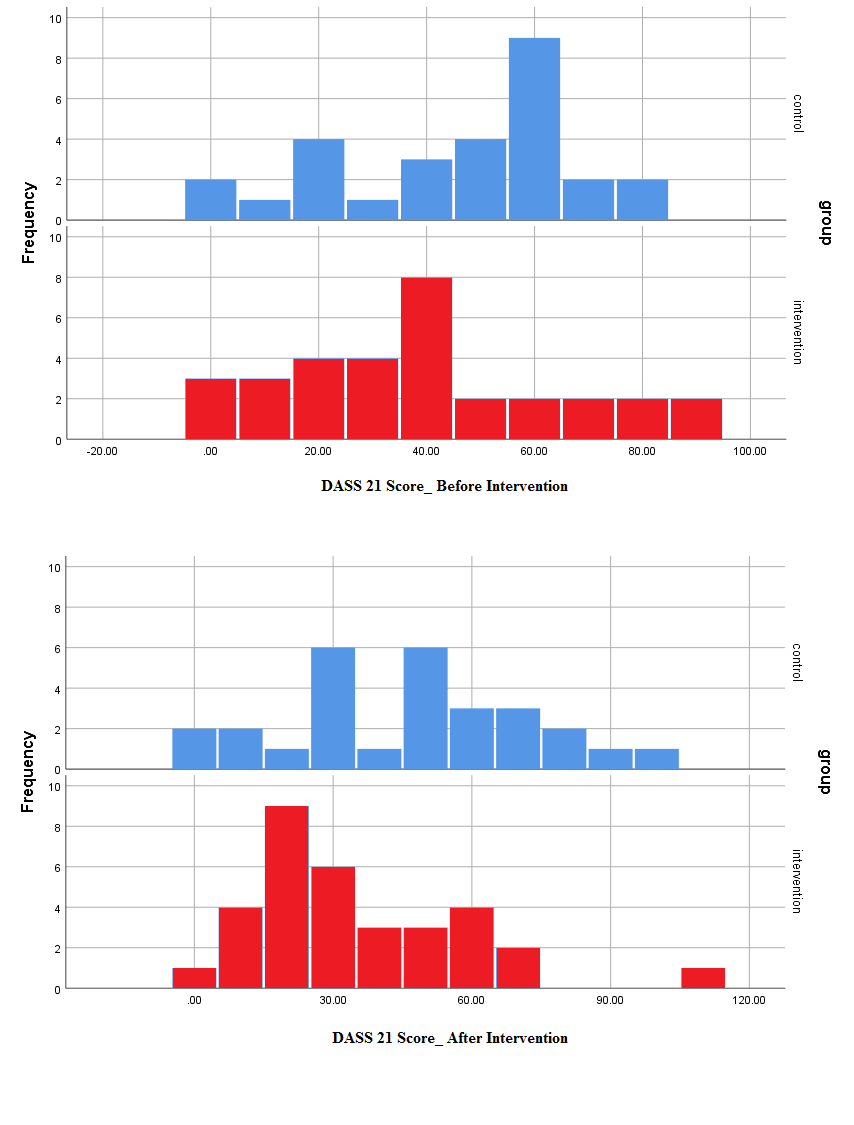
**
